# Posterior Cervical Fusion of Occiput-T3 for Unstable Complex Odontoid Fracture in an 80-Year-Old Male With C2-Sacrum Synostosis From Ankylosing Spondylitis: A Case Report

**DOI:** 10.7759/cureus.26897

**Published:** 2022-07-15

**Authors:** David Frolov, Matt Porter, Miguel Schmitz

**Affiliations:** 1 Orthopaedics/Spine, Elson S. Floyd College of Medicine, Washington State University, Spokane, USA; 2 Orthopaedics, Elson S. Floyd College of Medicine, Washington State University, Spokane, USA; 3 Orthopaedics, Alpine Orthopaedic & Spine, Spokane, USA; 4 Orthopaedics, Washington State University, Spokane, USA

**Keywords:** spinal surgery, long cervical fusion, posterior cervical fusion, dens fracture, odontoid fracture, cervical fracture, cervical synostosis, ankylosing spondylitis

## Abstract

Ankylosing spondylitis (AS) is an autoimmune arthritic condition that presents with inflammation of the axial skeleton and oligoarthritis of the peripheral joints. While its pathophysiology is not fully understood, the condition can lead to kyphosis and spontaneous intervertebral synostosis of the spine. AS is managed through both non-operative and operative means, but fractures in patients with AS are more complicated in those with synostosis. We present a case of a patient who is a tribal elder and Salish language instructor, with kyphotic AS with synostosis of C2-sacrum, and mobility confined to occiput-C1 and C1-C2. The patient suffered a low-energy fall backward from bed and presented to the orthopedic clinic approximately a month after his injury complaining of torticollis and neck pain. He was diagnosed to have a dens fracture, a right C2 pars/facet fracture, and a right lateral mass fracture with C1-C2 stenosis and cervical myelopathy. After the failure of conservative management, the patient required a full occiput-T3 fusion due to the osteoporosis and fragile AS synostosis of the spine, to mitigate transitional zone stresses that can occur with a shorter fusion. The fusion was successful, and it minimized the pain, corrected the torticollis, and allowed the patient to resume his tribal roles.

## Introduction

Ankylosing spondylitis (AS) is one of the most common seronegative spondyloarthropathies, and it primarily affects the spinal column and sacroiliac joints [1,2,3]. Occurring in 0.1-1.4% of the population, AS typically affects males, and becomes apparent between 20-30 years of age [2]. The pathophysiology of AS is poorly understood but is thought to be associated with the human leukocyte antigen B27 (HLA-B27) gene [3,4]. Ultimately, the immune response is initiated followed by ossification of the intervertebral joints, facet joints, and sacroiliac joints, forming bony deposits along the vertebral edges known as syndesmophytes. Synostosis occurs as the bony deposits of adjacent vertebrae fuse, resulting in the immobilization of the affected portion of the spine [3,4]. In most severe cases, progression of AS can lead to spontaneous intervertebral synostosis of the entire spine, forming a “bamboo-like” appearance on imaging [1,5,6].

Since AS is often associated with kyphosis and osteoporosis, the progression of the disease forms a long and brittle bony structure with a diminished ability to dissipate forces from trauma [1,2,5]. This increases the risk for spinal fractures with an increased odds ratio of 7.7 compared to the general population [7]. Fractures may even occur from low-energy trauma with hyperextension from ground-level falls being the most frequent mechanism of injury [1,2,8].

In patients with AS, the most common spinal fracture occurs in the cervical spine (53%), with an associated spinal cord injury occurring 27.5% of the time [7]. Hence, great care must be taken to prevent neurological deterioration from even seemingly trivial traumatic spinal injuries [7]. Conservative management has historically been the preferred course of treatment for cervical fractures [1]. However, recently, surgical alternatives have been considered more frequently as a surgical fixation offers the advantage of immediate immobilization, decreasing the risk of disease or fracture progression and neurological deterioration [6]. The surgical options include the anterior, posterior, or combined approach, though the posterior approach has been the preferred method as its outcomes are as favorable as those of the combined approach and superior to the anterior approach, while also leading to lesser complications [6,9]. Specifically, a long-segment posterior fixation is recommended along with the addition of a posterior bone graft [1,10]. An important consideration in posterior fixations is that the termination of the fusion should not end at the cervicothoracic junction but must extend to the thoracic spine, if necessary, to reduce the probability of incurring another fracture at this junction [6]. Postoperative management requires a molded cervical collar as the bone union can usually take up to three months to achieve [1,6].

## Case presentation

The patient was informed that the data concerning their case would be submitted for publication and gave permission for its use.

**Figure 1:**
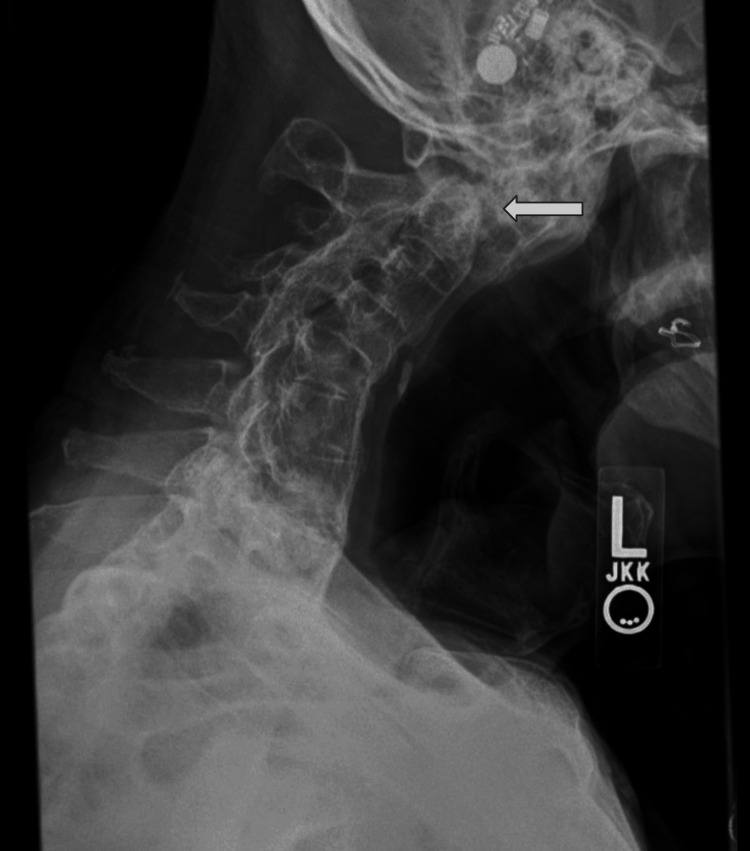
Preoperative lateral view X-ray of the dens fracture, bilateral C2 facet fracture, and lateral mass fractures with C1-C2 stenosis (white arrow) Note the dens fracture and ankylosed cervical spine and kyphosis

**Figure 2:**
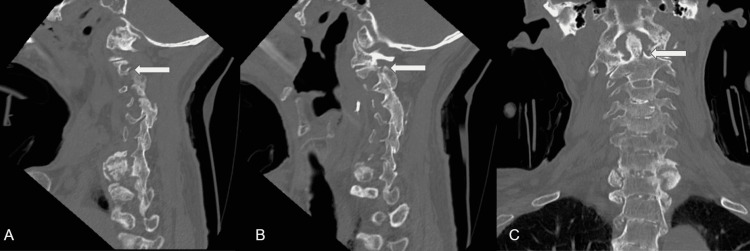
Left (A) and right (B) preoperative sagittal view CT scan of the facet fracture. Preoperative coronal view CT scan of the C2 dens/body fracture (C) CT: computed tomography

We report the case of an 80-year-old male with a history of kyphotic AS with synostosis of C2-sacrum, who presented with neck pain following a backward fall from a bed one month prior. He is a tribal elder and Salish language instructor hailing from a population with a higher prevalence of autoimmune disorders, specifically spondyloarthropathies [11]. He reported persisting neck pain and right-sided torticollis after the low-trauma fall. X-ray (Figure 1) and CT imaging (Figure 2) revealed a dens fracture, a right C2 facet fracture, and a right lateral mass fracture with C1-C2 stenosis.

**Figure 3:**
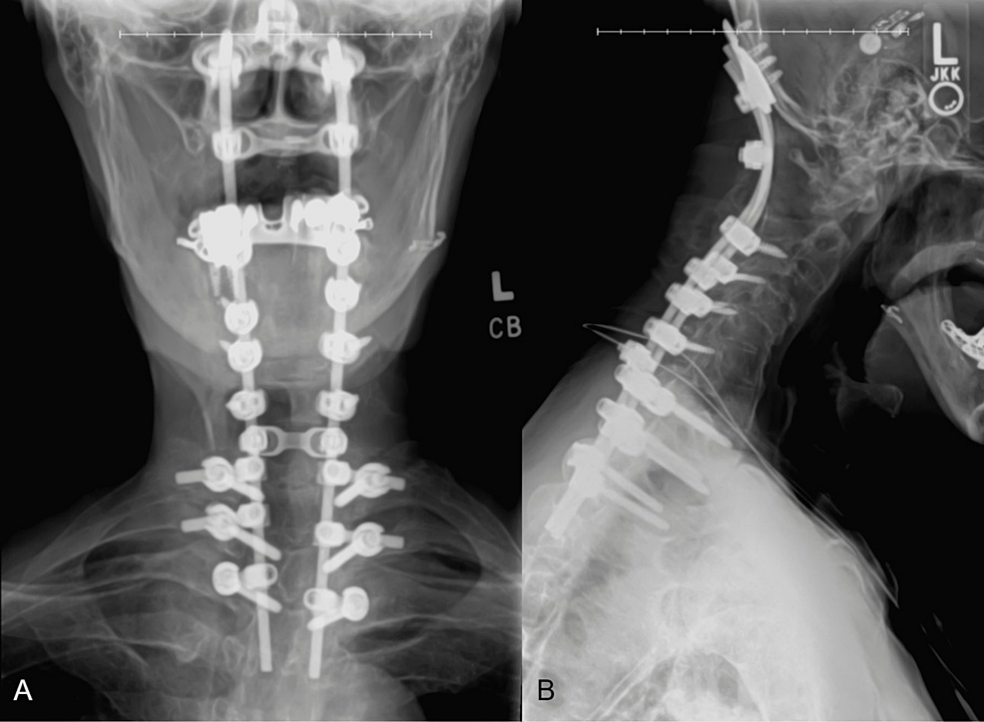
Coronal (A) and sagittal (B) views of postoperative CT scan of the posterior cervical fusion of occiput to T3 CT: computed tomography

There were spinal cord changes on MRI, but no neurological deficits were noted. As per the standard course of treatment, non-operative measures were first adopted, and the patient was placed in a cervical collar with hopes of achieving fibrosis stabilization or extension of the AS between the occiput and cervical spine to the extent that it would stabilize the fracture. Following three months of continued pain, the lifestyle imposition on the patient, ongoing torticollis, development of a non-functional fracture union, and spinal cord changes on MRI, the treating surgeon was compelled to continue with surgery. It was determined that the best course of action was to provide a posterior cervical fusion spanning the occiput to T3 as well as laminectomy of C1-C2 and occiput to C1 laminectomy/foramen magnum expansion (Figure 3).

Procedure

After providing general endotracheal anesthesia, neurological monitoring was applied, and the Mayfield head tongs were placed. The patient was very carefully rotated to the prone position upon an Amsco table. All pressure points were padded and were carefully monitored for proper positioning. The patient’s head was properly fixed to the Mayfield apparatus, which, in turn, was fixed to the table. After making an occiput to T4 incision, lateral mass screws were placed spanning C3-C6. Pedicle screws were placed from T1-T3. An occipital plate was placed and secured with 8-mm screws. C2 screws were not implemented as the patient had a fracture through the right C2 pars. C1 screws were not placed given the proximity of the C1 nerve root to the area where the C1 lateral mass screw would go and the fracture of the lateral mass on the right side. Afterward, 4-mm rods were placed and adjusted accordingly with acceptable alignment. Good fixation was obtained with the improved extension of his neck and reduction of the torticollis. A laminectomy was achieved about C1-C2 and occiput to C2. The Globus Medical Kinex synthetic bone matrix (Globus Medical, Audubon, PA) soaked in bone marrow aspiration was placed dorsal to the occiput through T3 and an autologous bone graft was applied dorsally to the Kinex after it was processed with a bone mill. Throughout the procedure, somatosensory evoked potentials (SSEPs), motor evoked potentials (MEPs), and free running electromyographies (EMGs) were utilized to confirm the acceptable function of the spinal cord.

Complications

After rotation into the prone position, the patient's neck was reduced with little to no traction. This caused the patient's cord signals including SSEPs and MEPs to go out. The traction was let off with an adjustment of the bed into less reverse Trendelenburg position and adjustment of the Mayfield tong apparatus. With the new position, spinal cord function returned to levels exhibited prior to the postural change.

After the procedure, the patient was noted to have acute blood-loss anemia that required a transfusion. After two weeks of rehabilitation, he was discharged to his remote home with his family without any further significant complications.

## Discussion

AS often progresses to kyphotic synostosis of the entire spine with associated osteoporosis, thereby increasing the risk of traumatic spinal fractures [1,2,5,7]. Our case specifically showed how a minor traumatic event concentrated forces at the last mobile joint in the cervical spine, resulting in a complex odontoid fracture.

The treatment of cervical fractures varies but often begins with conservative management [1,6]. For our patient specifically, a cervical collar was placed with the hope that he would develop a fibrosis stabilization or extension of the AS to stabilize the fracture. Surgical treatment was also deferred as this would require fusion of the patient’s last mobile vertebral joint. However, three months of non-operative management of the odontoid fracture proved to be unsuccessful. His pain persisted with associated torticollis. There was a continued threat to the spinal cord at the occiput/cervical junction from the nonunion of the fracture and spinal cord changes were seen on MRI. This compelled the treating surgeon to provide a posterior cervical fusion spanning the occiput to T3, to mitigate potential transitional zone stresses. Several considerations were made for the length of the fusion. Firstly, the patient was prone to having stress risers anywhere where there were kyphotic segments that did not have support, such as from the ribs. Second, he had osteoporosis deep to the shell of hard bone associated with the AS. Anecdotal experience with short fusions has shown that they are often associated with the loosening of screws with time. The brittle nature of the ankylosed cervical spine also increases the risk of low-trauma fractures, and with a fusion from the occiput, there was a concern for the risk of fracture at the termination of the surgical fusion [6,7]. Also, prior studies have shown that ending at the cervicothoracic junction increases the possibility of another fracture at this location [6]. And hence, it was determined to fuse caudally to T3 (Figure 3).

During the procedure, after the rotation of the patient into the prone position, his cord signals including SSEPs and MEPs went out. It was identified that there was originally a traction element to the patient's head that had occurred with the settling of the patient on the table during reverse Trendelenburg. The patient was taken out of reverse Trendelenburg and the traction was relaxed, resulting in the complete return of spinal cord functioning. Due to spinal fusion and loss of pliability in vertebral joints in AS patients, SSEPs, MEPs, and free-running EMGs are strongly encouraged during any spinal procedures to monitor for spinal cord damage and prevent neurological sequelae.

## Conclusions

The fusion was successful in our patient, minimizing pain and improving the position of the head. This allowed the patient to resume his tribal roles and drive autonomously. Extended follow-up identified the preservation of neurological status, reduction of the torticollis, fusion of the spine, and residual overall kyphosis of the cervical spine that was partially inevitable given the global kyphotic posture of the synostotic spine from the cervical spine through the sacral spine. Full occiput-T3 fusion appears to be a feasible option for C2 cervical fractures in patients with significant cervical synostosis from AS and osteoporosis.
